# Brachial plexopathy with Sjögren syndrome

**DOI:** 10.1002/ccr3.1510

**Published:** 2019-04-06

**Authors:** Sachin M. Bhagavan, Swathi Beladakere Ramaswamy, Gurpreet S. Khakh, Raghav Govindarajan

**Affiliations:** ^1^ Department of Neurology University of Missouri Health Care One Hospital Dr. CE507 Columbia 65212 Missouri

**Keywords:** Brachial plexopathy, brachial plexus, Sjögren syndrome, Sjögrens

## Abstract

This report will explain an unusual presentation of brachial plexopathy associated with manifestation of Sjögrens and will emphasize that Sjögrens may also present initially with neurological involvement only.

## Introduction

Sjögren syndrome can have a wide variety of neurological, both CNS (Central Nervous System) and PNS (Peripheral Nervous System) involvement. Here, we report a case of brachial plexopathy associated with Sjögren in a 22‐year‐old man who came with severe arm pain which later developed to arm and hand weakness. MRI of the brachial plexus showed patchy hyperintense T2 signal in the trunks. Further workup diagnosed Sjögren syndrome.

Brachial plexus involvement is commonly seen by physicians in both inpatient and outpatient consultations. Sjögren syndrome (SS) is an autoimmune condition that affects 0.01–0.1% of the adult population, and the disease overwhelmingly affects middle‐aged women 1 Neurologic involvement has been reported in primary SS in approximately 10–25% of cases 2, 3, most frequently as peripheral neuropathy 4. In this report, we describe brachial plexopathy in association with Sjogren's syndrome.

## Case

**Figure 1:**
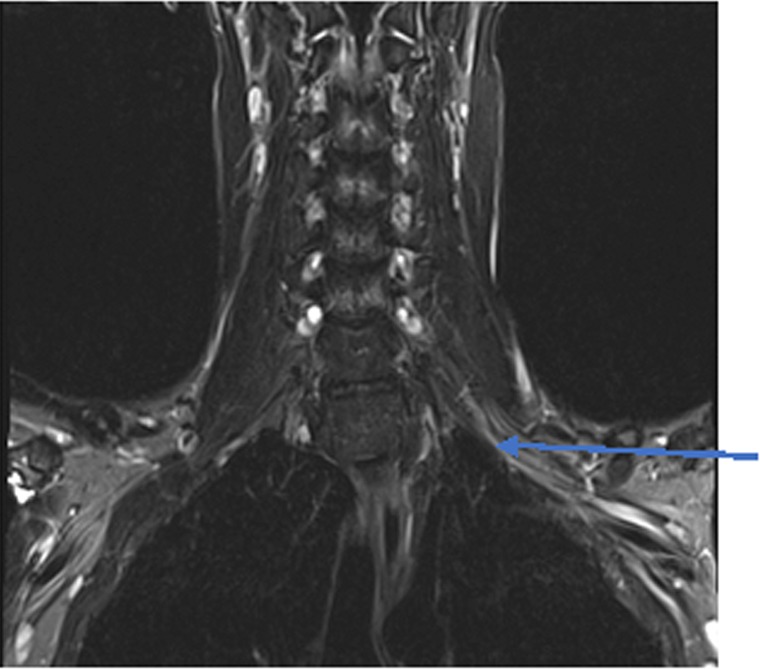
MRI brachial plexus with and without contrast. T2 coronal section shows patchy T2 hyperintensity of the left brachial plexus affecting all the trunks (arrow).

A 22‐year‐old man woke up with severe left shoulder pain which later became a severe arm pain. He was diagnosed with shoulder sprain (as noted by his referring physician) initially and was put in a sling. There was no evidence of trauma. Despite these measures, the pain persisted, and he was prescribed multiple medications including paracetamol, gabapentin 600 mg three times a day and hydrocodone 5 mg three times a day PRN. Two weeks later, he started developing hand and arm weakness at which time he was evaluated by us. On examination, he had 2/5 wrist extension, 3/5 finger flection, 2/5 abduction of fifth digit, and 4/5 shoulder abduction but no scapular winging. Pin prick testing did not show sensory loss along the dermatomes. Electrodiagnostic study performed a month from onset showed denervation in multiple muscles innervated by median, ulnar, radial, and axillary nerves. MRI of the brachial plexus with and without contrast showed patchy T2 hyperintense signal involving all the trunks of the left brachial plexus (Fig. 1). There was no enhancement with contrast. He was diagnosed with idiopathic brachial plexopathy (neuralgic amyotrophy) and managed conservatively with physical therapy and gabapentin 300 mg three times a day for neuropathic pain. Two months later, he complained of dry eyes and dry mouth. Hence, further workup was performed which showed antinuclear antibodies at 1:1200 (Mayo Clinic, Normal <1:40) and a positive SSA antibody 3.5U (Mayo Clinic, normal‐ <1.0U). See Table 1. Lip biopsy showed focal lymphocytic sialadenitis of the minor salivary glands confirming the diagnosis of Sjögren syndrome (based on American College of Rheumatology criteria). He was treated with in hydroxychloroquine in addition to intravenous immunoglobulin (induction dose‐2 g/kg actual body weight split over 5 days followed by 1 g/kg actual body weight split over 2 days every 6 weeks for a total of eight doses) for brachial plexopathy.

**Table 1:** Depicts the autoimmune panel of the patient

Antibody panel	Test result
RNP Antibody	Negative
SM Antibody	Negative
SS‐A Antibody	Positive (A)
SSB/La	Negative
SCL70	Negative
Ds DNA	Negative
FANA	Positive (A)
FANA titre	1:1280
FANA pattern	Speckled
FANA interpretation	Positive
ANCA	Negative

ANCA, antineutrophil cytoplasmic antibody; Ds DNA, double‐strand deoxyribonucleic acid; FANA, fluorescent antinuclear antibody; RNP, ribonucleoprotein; SCL, scleroderma; SM, anti‐Smith; SSA, anti‐Sjögren's syndrome‐related antigen A; SSB, anti‐Sjögren's syndrome‐related antigen B.

## Discussion

Sjögren syndrome is a chronic autoimmune disease involving lymphocytic infiltration of exocrine glands associated with production of various autoantibodies in the blood. According to commonly used criteria for diagnosing Sjogren, the AECG (American‐European Consensus Group criteria for Sjögrens) and ACR (American College of Rheumatology) criteria do not include neurological manifestations for diagnosis. Therefore, in majority of patients presenting with neurological manifestations, there is usually a low clinical suspicion for Sjögrens.

**Figure 2:**
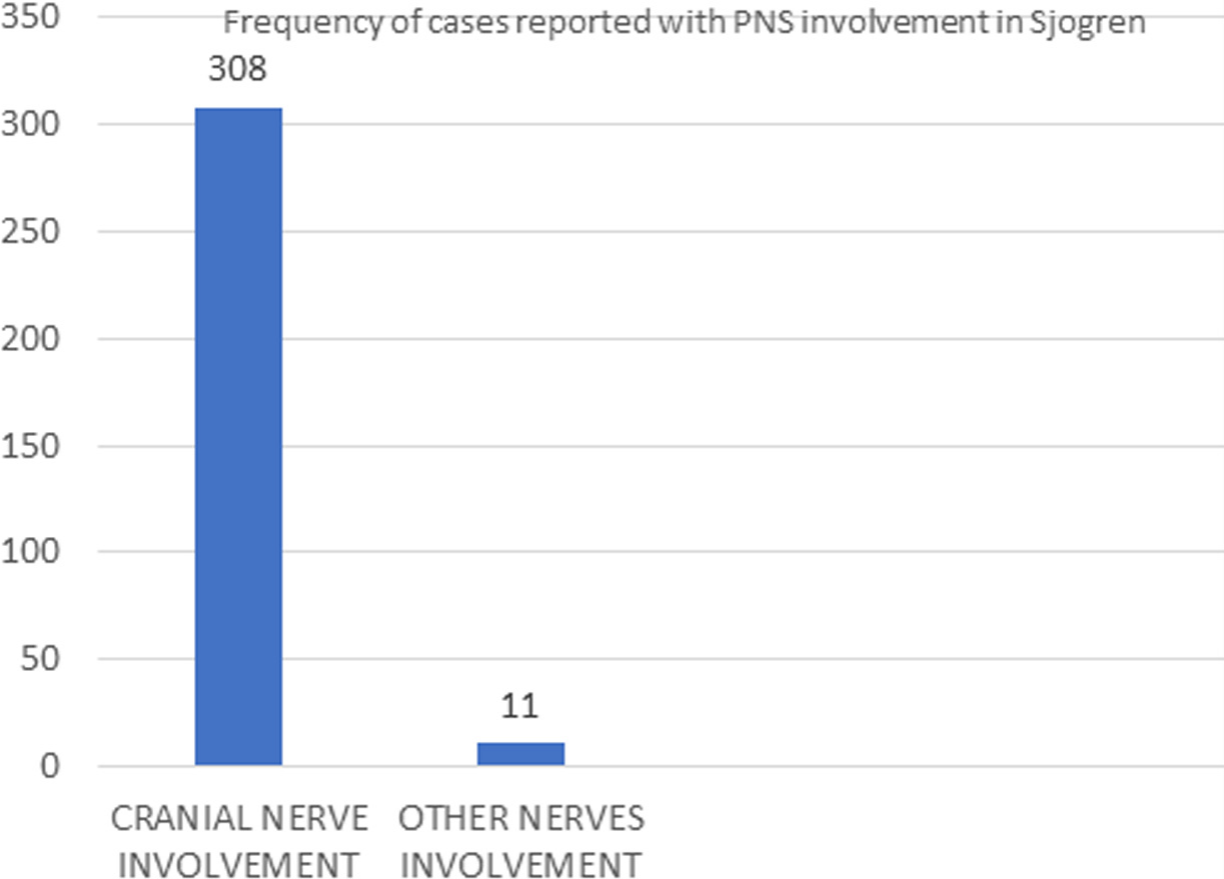
Contrasts the number of cases reported worldwide involving cranial nerves versus other nerves involvement. Note the marked number of cases with cranial nerve involvement with respect to other nerves 6.

**Figure 3:**
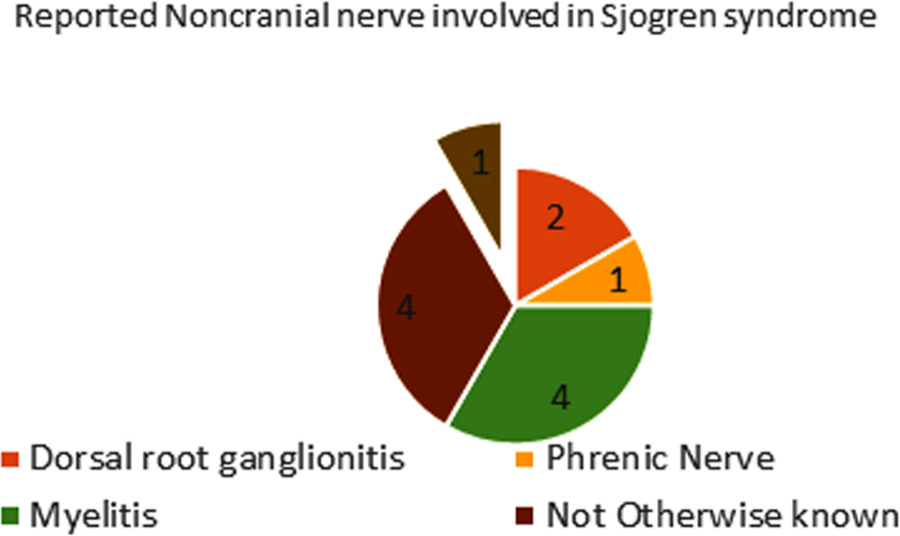
States the number of reported cases with different noncranial nerves involved in Sjogren 6.

Peripheral nervous system involvement is seen occurring in 10–20% of patients with Sjögren syndrome 5 and appears in various forms, such as sensory neuropathy, multiple mononeuropathy, multiple cranial neuropathy, autonomic neuropathy, and radiculoneuropathy 5, 6. Rapidly progressive brachial diplegia has also been reported with Sjogren syndrome 7. Figures 2 and 3 depict the number of reported cases with focal involvement of peripheral nervous system with Sjogren syndrome 6.

In this case, the presentation of left shoulder pain and subsequent hand and arm weakness without any inciting factor lead to an initial impression of Parsonage–Turner syndrome (idiopathic brachial neuritis) which usually presents with asymmetric involvement of the brachial plexus 8, 9 in the form of acute or subacute shoulder girdle 10 and arm pain, followed by weakness and often wasting of the muscles 9. But later development of exocrine deficient symptoms and subsequent diagnosis of Sjögrens led to the possibility of these neurological manifestations being a part of Sjögrens.

## Conclusion

Brachial plexopathy can be associated with Sjogren's syndrome; therefore, a thorough evaluation of the patient for secondary causes can be considered in those cases especially when sicca symptoms are reported**.**

## Authorship

SMB: involved in concepts design, literature search, data acquisition, data analysis, and manuscript preparation. SBR: performed literature search, data acquisition, data analysis, and manuscript preparation. GSK: involved in concepts design, literature search, data acquisition, data analysis, and manuscript review. RG:designed concepts, searched literature, involved in data acquisition, data analysis, and manuscript review, Guarantor.

## Conflict of Interest

None declared.
